# Gastrointestinal permeability in ovarian cancer and breast cancer patients treated with paclitaxel and platinum

**DOI:** 10.1186/1471-2407-7-155

**Published:** 2007-08-09

**Authors:** Bohuslav Melichar, Radomír Hyšpler, Emanuela Dragounová, Josef Dvořák, Hana Kalábová, Alena Tichá

**Affiliations:** 1 Department of Oncology & Radiotherapy, Charles University Medical School & Teaching Hospital, Sokolská 581, Building 23, 500 05, Hradec Králové, Czech Republic; 2 Department of Gerontology & Metabolic Care, Charles University Medical School & Teaching Hospital, Sokolská 581, Building 22, 500 05, Hradec Králové, Czech Republic; 3 Department of Gynecology & Obstetrics, Charles University Medical School & Teaching Hospital, Sokolská 581, 500 05, Hradec Králové, Czech Republic

## Abstract

**Background:**

Combination of platinum derivatives with paclitaxel is currently the standard front line regimen for patients with epithelial ovarian carcinoma, and represents also an active regimen in patients with metastatic breast or unknown primary carcinomas. Measurement of intestinal permeability represents one of the potential methods of noninvasive laboratory assessment of gastrointestinal mucositis induced by chemotherapy, but little is known about intestinal permeability in patients treated with paclitaxel or platinum.

**Methods:**

Intestinal permeability was assessed in 36 breast and ovarian cancer patients treated with paclitaxel/platinum combination by measuring, using capillary gas chromatography, urinary sucrose, lactulose, xylose and mannitol after oral challenge. The significance of differences during the therapy compared to pre-treatment values was studied by Wilcoxon paired test. The differences between groups of patient were studied by Mann-Whitney U test. Fisher exact test was used to compare the frequency in different subgroups.

**Results:**

After administration of the first dose, a significant (p < 0.05) decrease in xylose absorption and increased lactulose/mannitol, sucrose/mannitol, lactulose/xylose and sucrose/xylose ratios were observed, but these parameters returned subsequently to pre-treatment levels. Patients who experienced serious (grade 3 or 4) toxicity had at baseline significantly lower percentages of xylose, mannitol and sucrose, and higher lactulose/mannitol ratio. Nine of 13 (69%) patients with baseline lactulose/mannitol ratio 0.070 or above experienced serious toxicity compared to 4 out of 23 patients (17%) with the ratio below 0.070 (p = 0.002). Post-treatment lactulose, lactulose/mannitol, sucrose/mannitol and lactulose/xylose ratios were significantly increased in patients with serious toxicity.

**Conclusion:**

A transient significant increase in lactulose/monosaccharide and sucrose/monosaccharide ratios was observed in ovarian and breast cancer patients treated with paclitaxel and platinum. Increased lactulose absorption, lactulose/mannitol, sucrose/mannitol and lactulose/xylose ratios were evident in patients with grade 3 or 4 toxicity, and increased baseline lactulose/mannitol ratio predicted serious toxicity.

## Background

Combination of platinum derivatives (cisplatin or carboplatin) with paclitaxel is currently the standard front line regimen for patients with advanced epithelial ovarian carcinoma (EOC) after demonstration of superior survival in randomized clinical trials [1,2]. This combination is also highly active in patients with metastatic breast carcinoma [3], or in patients with unknown primary carcinoma [4].

Hematologic toxicity (neutropenia, thrombocytopenia and anemia) is the most important side effect of paclitaxel/platinum combination, but, similarly to many other regimens of cytotoxic chemotherapy, gastrointestinal mucositis is common in patients treated with paclitaxel/platinum. The term gastrointestinal mucositis is used to describe the damage of mucosa distal to oral cavity induced by cytotoxic drugs or radiation [5,6]. Any part of the gastrointestinal tract from the oral cavity to the anus may be affected. Depending on the region of gastrointestinal tract affected, gastrointestinal mucositis may manifest as dysphagia, dyspepsia, diarrhea, abdominal cramping, or rectal bleeding. The incidence of severe gastrointestinal mucositis in patients treated with taxane/platinum combination is around 15% [1,7], but clinical manifestations of gastrointestinal mucositis of milder degree are found in most patients treated with paclitaxel/platinum. In addition, management of gastrointestinal mucositis may be a problem in EOC patients with gastrointestinal function compromised as a consequence of involvement of peritoneal cavity by the tumor, or ablative surgery.

While mucosal damage in the oral cavity can be easily assessed by direct inspection, involvement of other regions of the gastrointestinal tract may be evaluated solely by endoscopy, which is less easily performed in a patient coping with the side effects of anti-cancer therapy. The diagnosis and the assessment of severity of gastrointestinal mucositis therefore still rely on anamnestic data.

Intestinal permeability is used to study the disorders of gut mucosa in benign disorders, such as inflammatory bowel disease and gluten enteropathy [8,9]. The measurement of absorption of biologically inert sugars has been used for the estimation of intestinal permeability in most studies, usually combining a disaccharide and a monosaccharide with the results of differential excretion expressed as a ratio. Under physiological conditions, monosaccharides are readily absorbed by the intestinal villi, while disaccharides that are absorbed in the crypt epithelium are excluded. Atrophy of the villi may result in the decrease in monosaccharide absorption and increased exposure of the crypts to luminal contents. The dysfunction of small bowel mucosa is therefore characterized by an increase in disaccharide/monosaccharide (e.g. lactulose/mannitol) ratio. In addition to the assessment of the function of small intestine, dysfunction of gastroduodenal mucosa may be assessed using sucrose, a disaccharide normally digested in the small intestine [10]. Intestinal permeability has also been investigated in patients treated with cytotoxic drugs. Alterations of intestinal permeability similar to those found in patients with benign intestinal disorders were found after administration of different cytotoxic agents, including fluoropyrimidines, alkylating compounds and anthracyclines [11], but there is virtually no information on intestinal permeability in patients treated with a taxane/platinum combination.

In the present study, we investigated intestinal permeability in ovarian and breast cancer patients treated with paclitaxel and platinum chemotherapy.

## Methods

### Patients

A total of 36 women, mean (± standard deviation) age 57 ± 11 (range 34 – 79) years, treated with paclitaxel and platinum derivatives were included in the present study. Fifteen patients had ovarian cancer (primary EOC 13 patients and Krukenberg tumors 2 patients), 19 patients had breast carcinoma and 2 patients had adenocarcinoma of unknown primary (Table 1). Five patients with primary EOC also had a history of breast cancer. Ten patients were chemotherapy-naïve and 26 patients had a history of previous chemotherapy. Twenty-one patients were treated with the combination of paclitaxel (175 mg/m^2 ^for 1 to 3 hours) and carboplatin (area under the curve 6) administered every 3 weeks. One patient with carboplatin allergy received the combination of paclitaxel (175 mg/m^2^) and cisplatin (75 mg/m^2^). Thirteen patients were treated with a weekly regimen of paclitaxel (90 mg/m^2^) with carboplatin (area under the curve 2) with (9 patients) or without trastuzumab (4 mg/kg loading dose, then 2 mg/kg weekly; 4 patients), and one patient, who was originally considered for the combination chemotherapy with carboplatin, was subsequently treated with the weekly regimen of paclitaxel and trastuzumab. The toxicity was assessed using Common Terminology Criteria for Adverse Events version 3.0 [12]. Serious toxicity was defined as of grade 3 or higher. The protocol was approved by the University Hospital Hradec Králové Ethics Committee, and the patients signed informed consent.

**Table 1:** Characteristics of patients

**patient**	**age (years)**	**diagnosis**	**previous chemotherapy**	**regimen**	**timing of measurement after the first dose (days from start)**	**toxicity during first 6 weeks of therapy (grade)**	**baseline lactulose/mannitol ratio**	**lactulose/mannitol ratio after the first dose**
1	52	EOC (history of BC)	yes	q3w (cisplatin)	6	diarrhea (2)	0.069	0.033
2	66	EOC	none	q3w	7	diarrhea (1)	0.030	0.011
3	67	EOC (history of BC)	none	q3w	7	none	0.096	0.047
4	46	EOC	yes	q3w	7	**neutropenia (3)**	0.027	0.079
5	52	EOC	none	q3w	7	none	0.017	0.017
6	39	unknown primary	none	q3w	6	none	0.044	0.021
7	39	Krukenberg	yes	q3w	7	**diarrhea (3)**	0.111	0.132
8	62	EOC (history of BC)	yes	q3w	4	none	0.090	0.018
9	71	EOC	yes	q3w	7	**neutropenia (4)**	0.108	0.770
10	67	EOC	none	q3w	7	leukopenia (2)	0.061	0.129
11	64	BC	yes	q3w	7	leukopenia (2)	0.041	0.144
12	56	EOC	yes	q3w	7	none	0.031	0.027
13	64	BC	yes	q3w	7	none	0.041	0.032
14	58	BC	yes	q3w	7	diarrhea (1)	0.026	0.132
15	60	BC	yes	q3w	7	none	0.033	0.054
16	45	BC	yes	q3w	7	none	0.033	0.110
17	54	BC	yes	q3w	7	**nausea (3)**	0.184	0.438
18	58	BC	yes	q3w	7	**neutropenia (3)**	0.038	0.057
19	61	unknown primary	none	q3w	6	diarrhea (2)	0.055	0.063
20	66	BC	yes	q3w	2	leukopenia (2)	0.031	0.040
21	46	EOC (history of BC)	none	q3w	7	none	0.047	0.029
22	52	EOC (history of BC)	yes	q3w	7	leukopenia (2)	0.063	0.119
23	59	BC	yes	weekly P+C+T	7	**anemia (3)**	0.072	0.094
24	78	BC	yes	weekly P+ T	7	**diarrhea (3)**	0.094	0.133
25	45	BC	yes	weekly P+C+T	7	none	0.069	0.128
26	63	BC	none	weekly P+C	7	none	0.075	0.060
27	36	Krukenberg	yes	weekly P+C	7	**leukopenia (3)**	0.072	0.106
28	57	BC	yes	weekly P+C+T	7	none	0.038	0.041
29	74	EOC	none	weekly P+C	2	**nausea (3)**	0.090	0.130
30	41	BC	yes	weekly P+C+T	22	**neutropenia (4); stomatitis (3)**	0.038	0.030
31	79	EOC	none	weekly P+C	3	**nausea (3)**	0.120	0.118
32	34	BC	yes	weekly P+C+T	7	none	0.025	0.010
33	63	BC	yes	weekly P+C+T	8	**diarrhea (3)**	0.075	0.070
34	55	BC	yes	weekly P+C+T	7	**neutropenia (3)**	0.043	0.069
35	62	BC	yes	weekly P+C+T	7	none	0.082	0.099
36	54	BC	yes	weekly P+C+T	7	none	0.030	0.044

Grade 3 or 4 toxicity is highlighted by bold type. BC breast carcinoma; C carboplatin; EOC epithelial ovarian carcinoma; q3w paclitaxel/carboplatin every 3 weeks (cisplatin in patient 1); P paclitaxel; T trastuzumab

### Measurement of intestinal permeability

Intestinal permeability was studied by measuring urinary sucrose, lactulose, xylose and mannitol after oral challenge [13]. After an overnight fast, a pre-test urine sample was collected to detect any endogenous mannitol and the patients ingested 100 ml of the test solution containing 2 g of mannitol, 2 g of xylose, 10 g of lactulose, and 25 g of sucrose in water. The patients then continued fasting for 2 hours, and urine was collected for 5 hours. Urine samples were stored at -24°C until analysis.

Lactulose, xylose, sucrose and mannitol were determined by capillary gas chromatography as described [13], and urinary excretion was expressed as percentage of the ingested dose as well as the ratio of lactulose/mannitol, sucrose/mannitol, lactulose/xylose and sucrose/xylose. All chemicals were obtained from Sigma (St. Louis, MO, USA). Briefly, samples were thawed, spiked with internal standard (phenyl-β-D-glucoside) and evaporated under vacuum to dryness (Eppendorf Concentrator 5301, Eppendorf, Hamburg, Germany). The dried residues were derivatized by hydroxylamine in pyridine (75°C for 30 minutes) and bis(trimethylsillyl)trifluoracetamide (75°C for 15 minutes). The aliquot of the sample was injected into gas chromatograph equipped with a non-polar column and flame ionization detector (GC 8000, Fisons Instruments, Milano, Italy). Chromatographic data were collected using Clarity software (DataApex, Prague, Czech Republic). The method is specific as no interfering peaks were found, and other mono- and disaccharides did not interfere with the sugars used for gastroduodenal and intestinal permeability testing. The detection limit (10 mg/l) and quantitation limit (50 mg/l) were identical for mannitol, xylose, sucrose and lactulose, and reproducibility calculated as coefficient of variation was consistently below 7%. The quality control was performed by the measurement of customized stored quality control samples along with each set of specimens.

The test was performed before the start of therapy (baseline), during the first cycle of chemotherapy (one week after the start of treatment), at the end of the first or second cycle of chemotherapy, and before subsequent chemotherapy cycles.

### Statistical analysis

The significance of differences during the therapy compared to pre-treatment values was studied by Wilcoxon paired test. The differences between groups of patients were studied by Mann-Whitney U test. Serial measurements were evaluated using the means of subsequent measurements as described [14], and these means of measurements were compared with baseline values by Wilcoxon paired test. Fisher exact test was used to compare the frequency in different subgroups. The analyses were performed using NCSS 2001 software (Number Cruncher Statistical Systems, Kaysville, Utah, USA). The decision on statistical significance was based on p = 0.05 level.

## Results

### Intestinal permeability during the therapy with paclitaxel/platinum

No significant differences were observed at baseline in the parameters of intestinal permeability between patients with ovarian cancer, and breast or unknown primary carcinomas, with the exception of lower percentage of mannitol absorption in ovarian cancer patients (mean ± standard error of the mean 8.7 ± 1.9% vs. 13.3 ± 1.6%, p = 0.01). Similarly, no significant differences were observed between chemotherapy-naïve patients and patients who had previous chemotherapy.

**Figure 1:**
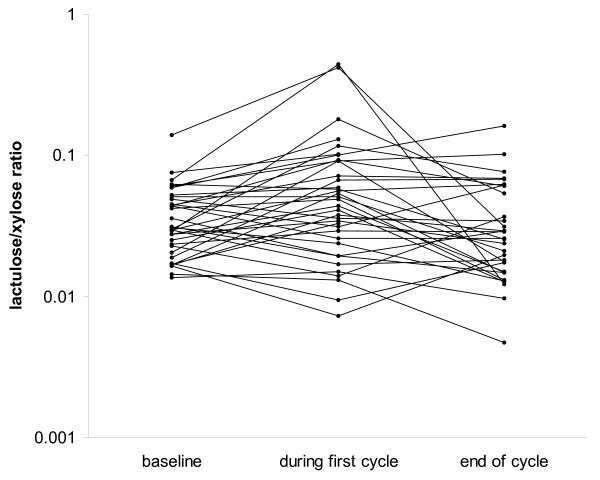
**Lactulose/xylose ratio before and during therapy**. Urinary lactulose and xylose were determined after oral challenge before the treatment, during the first cycle and at the end of first or second cycle. Shown are results of measurements obtained in individual patients.

In all patients, intestinal permeability was assessed during the first cycle, 7 ± 3 days after the first dose of chemotherapy. At this time point, a decrease in xylose absorption, an increase in sucrose absorption of borderline significance and significantly increased lactulose/mannitol, sucrose/mannitol, lactulose/xylose (Figure 1), and sucrose/xylose ratios were observed. Subsequently, at least one additional measurement was obtained in 32 patients. The reason for no subsequent measurements in the remaining 4 patients was interruption of chemotherapy in 3 cases and technical problems with sample collection in one case. The median number of these additional measurements was 3 (range 1 – 7). The earliest next measurement was obtained 24 ± 9 days after the start of therapy (at the end of the first or second chemotherapy cycle). At this time, all parameters of intestinal permeability were not significantly different from baseline (Table 2). Similarly, the means of all measurements subsequent to the measurement in the first cycle were not significantly different from the baseline. The changes in intestinal permeability were similar in patients treated with weekly or 3-week regimen with the exception of percentage of xylose absorption that remained unchanged in patients treated with weekly regimen.

**Table 2:** Intestinal permeability before and during the therapy

**parameter**	**baseline (before therapy)** **(mean ± SEM)** **(95% CI)** (range)	**after the first dose** **(mean ± SEM)** **(95% CI)** (range)	**p (compared to baseline)**	**mean of subsequent measurements** **(mean ± SEM)** **(95% CI)** (range)	**p (compared to baseline)**	**at the end of first or second cycle** **(mean ± SEM)** **(95% CI)** (range)	**p (compared to baseline)**
xylose (%)	**17.1 ± 1.6** **(13.9 – 20.2)** (0.8 – 45.3)	**13.5 ± 1.5** **(10.5 – 16.4)** (2.2 – 40.7)	0.03	**16.2 ± 1.1** **(14.0 – 18.5)** (4.2 – 35.5)	0.40	**17.1 ± 1.6** **(13.8 – 20.3)** (2.4 – 53.2)	0.41
mannitol (%)	**11.4 ± 1.3** **(8.8 – 14.0)** (0.5 – 34.2)	**9.6 ± 0.9** **(7.7 – 11.4)** (1.4 – 24.6)	0.21	**13.1 ± 1.3** **(10.4 – 15.9)** (2.0 – 36.2)	0.58	**10.5 ± 1.0** **(8.4 – 12.6)** (1.1 – 23.9)	0.16
sucrose (%)	**0.41 ± 0.06** **(0.30 – 0.52)** (0.05 – 1.40)	**0.54 ± 0.07** **(0.39 – 0.70)** (0.06 – 1.71)	0.06	**0.47 ± 0.08** **(0.30 – 0.64)** (0.08 – 2.51)	0.73	**0.47 ± 0.09** **(0.28 – 0.65)** (0.07 – 2.51)	0.43
lactulose (%)	**0.57 ± 0.07** **(0.42 – 0.73)** (0.06 – 2.40)	**0.64 ± 0.07** **(0.49 – 0.78)** (0.05 – 2.08)	0.46	**0.56 ± 0.08** **(0.41 – 0.72)** (0.12 – 2.19)	0.49	**0.59 ± 0.10** **(0.39 – 0.78)** (0.07 – 2.19)	0.30
lactulose/mannitol ratio	**0.061 ± 0.006** **(0.049 – 0.073)** (0.017 – 0.184)	**0.101 ± 0.023** **(0.055 – 0.147)** (0.010 – 0.770)	0.02	**0.059 ± 0.006** **(0.047 – 0.070)** (0.017 – 0.143)	0.54	**0.058 ± 0.006** **(0.045 – 0.071)** (0.009 – 0.136)	0.77
sucrose/mannitol ratio	**0.038 ± 0.004** **(0.030 – 0.046)** (0.008 – 0.098)	**0.077 ± 0.017** **(0.043 – 0.111)** (0.012 – 0.585)	0.004	**0.048 ± 0.006** **(0.035 – 0.061)** (0.008 – 0.154)	0.16	**0.044 ± 0.006** **(0.031 – 0.056)** (0.009 – 0.152)	0.37
lactulose/xylose ratio	**0.039 ± 0.004** **(0.030 – 0.047)** (0.014 – 0.139)	**0.074 ± 0.016** **(0.042 – 0.107)** (0.007 – 0.443)	0.004	**0.041 ± 0.006** **(0.027 – 0.054)** (0.008 – 0.166)	0.69	**0.037 ± 0.006** **(0.025 – 0.049)** (0.004 – 0.162)	0.90
sucrose/xylose ratio	**0.024 ± 0.002** **(0.019 – 0.029)** (0.004 – 0.061)	**0.057 ± 0.011** **(0.035 – 0.080)** (0.009 – 0.336)	0.001	**0.034 ± 0.007** **(0.020 – 0.048)** (0.004 – 0.186)	0.31	**0.029 ± 0.006** **(0.017 – 0.041)** (0.003 – 0.186)	0.74

Shown is the mean ± standard error of the mean (SEM) of respective parameters. 95% confidence intervals (CI) of the mean are highlighted by the bold type, and range is indicated in the parenthesis below.

### Intestinal permeability and toxicity

A total of 13 patients experienced a serious toxicity (grade 3 or 4 adverse events) during the first 6 weeks of therapy (grade 3 or 4 leukopenia or neutropenia 6 patients, grade 3 diarrhea 3 patients, grade 3 nausea 3 patients, and grade 3 anemia 1 patient). Compared to other patients, patients who experienced serious toxicity had significantly lower percentages of xylose, mannitol and sucrose, and higher lactulose/mannitol ratio at baseline (Table 3). In addition, post-treatment lactulose, lactulose/mannitol, sucrose/mannitol and lactulose/xylose ratios were significantly increased in patients with serious toxicity. Thirteen patients had baseline lactulose/mannitol ratio 0.070 (95% upper confidence limit of the mean of all patients) or above. Nine of these patients (69%) experienced serious toxicity compared to 4 out of 23 patients (17%) with lactulose/mannitol ratio below 0.070 (p = 0.003).

**Table 3:** Comparison between intestinal permeability before therapy and after the first cycle in patients with or without serious toxicity

**parameter**	**no serious toxicity** **(n = 23)** **(mean ± SEM)** **(95% CI)** (range)	**serious toxicity** **(n = 13)** **(mean ± SEM)** **(95% CI)** (range)	**p**
baseline xylose (%)	**19.2 ± 2.0** **(15.2 – 23.3)** (2.6 – 45.3)	**13.2 ± 2.4** **(8.0 – 18.3)** (0.8 – 30.4)	0.05
baseline mannitol (%)	**13.5 ± 1.7** **(10.0 – 17.0)** (1.8 – 34.2)	**7.6 ± 1.4** **(4.6 – 10.7)** (0.5 – 18.1)	0.02
baseline sucrose (%)	**0.47 ± 0.07** **(0.33 – 0.62)** (0.05 – 1.40)	**0.30 ± 0.09** **(0.11 – 0.49)** (0.05 – 1.17)	0.05
baseline lactulose (%)	**0.60 ± 0.10** **(0.40 – 0.80)** (0.11 – 2.40)	**0.53 ± 0.12** **(0.28 – 0.78)** (0.06 – 1.50)	0.26
baseline lactulose/mannitol ratio	**0.049 ± 0.005** **(0.039 – 0.059)** (0.017 – 0.096)	**0.082 ± 0.012** **(0.056 – 0.108)** (0.027 – 0.184	0.01
baseline sucrose/mannitol ratio	**0.038 ± 0.004** **(0.029 – 0.047)** (0.008 – 0.080)	**0.038 ± 0.007** **(0.023 – 0.053)** (0.013 – 0.098)	0.92
baseline lactulose/xylose ratio	**0.032 ± 0.003** **(0.026 – 0.039)** (0.014 – 0.063)	**0.050 ± 0.009** **(0.030 – 0.069)** (0.017 – 0.139)	0.10
baseline sucrose/xylose ratio	**0.025 ± 0.003** **(0.019 – 0.031)** (0.006 – 0.058)	**0.023 ± 0.004** **(0.014 – 0.033)** (0.004 – 0.061)	0.64
xylose after the first dose (%)	**12.9 ± 1.6** **(9.5 – 16.2)** (3.7 – 34.5)	**14.5 ± 3.0** **(8.1 – 21.0)** (2.2 – 40.7)	0.82
mannitol after the first dose (%)	**10.1 ± 1.2** **(7.6 – 12.6)** (1.4 – 24.6)	**8.6 ± 1.5** **(5.4 – 11.7)** (2.1 – 19.2)	0.45
sucrose after the first dose (%)	**0.46 ± 0.08** **(0.28 – 0.63)** (0.06 – 1.71)	**0.70 ± 0.14** **(0.41 – 1.00)** (0.10 – 1.60)	0.12
lactulose after the first dose (%)	**0.50 ± 0.08** **(0.34 – 0.67)** (0.05 – 1.67)	**0.87 ± 0.12** **(0.62 – 1.13)** (0.50 – 2.08)	0.004
lactulose/mannitol ratio after the first dose	**0.061 ± 0.009** **(0.042 – 0.081)** (0.010 – 0.144)	**0.171 ± 0.057** **(0.047 – 0.300)** (0.030 – 0.770)	0.008
sucrose/mannitol ratio after the first dose	**0.052 ± 0.009** **(0.034 – 0.069)** (0.012 – 0.148)	**0.123 ± 0.042** **(0.032 – 0.214)** (0.020 – 0.585)	0.03
lactulose/xylose ratio after the first dose	**0.050 ± 0.009** **(0.031 – 0.068)** (0.007 – 0.180)	**0.118 ± 0.039** **(0.032 – 0.200)** (0.014 – 0.443)	0.04
sucrose/xylose ratio after the first dose	**0.042 ± 0.008** **(0.026 – 0.058)** (0.009 – 0.124)	**0.085 ± 0.027** **(0.027 – 0.143)** (0.010 – 0.336)	0.19

A total of 13 patients experienced a serious toxicity (grade 3 or 4 adverse events) during the first 6 weeks of therapy (grade 3 or 4 leukopenia 6 patients, grade 3 diarrhea 3 patients, grade 3 nausea 3 patients, and grade 3 anemia 1 patient). Shown is the mean ± standard error of the mean (SEM) of respective parameters. 95% confidence intervals (CI) of the mean are highlighted by the bold type, and range is indicated in the parenthesis below.

## Discussion

Intestinal permeability had been previously studied in cancer patients treated with different chemotherapy regimens [15-19], and the present study extends the observations of alterations of intestinal permeability in patients treated with anthracyclines, fluoropyrimidines and alkylating agents to paclitaxel and platinum chemotherapy. The present study included patients with ovarian cancer, breast cancer and unknown primary carcinoma treated with different regimens of paclitaxel/platinum chemotherapy, but no significant differences in gastrointestinal permeability were observed based on primary or regimen of chemotherapy, and statistical analyses were performed for the group as a whole. The rate of severe gastrointestinal mucositis induced by paclitaxel/platinum combination chemotherapy is relatively low compared to the incidence of myelosuppression in this patient population. Grade 3 or 4 gastrointestinal mucositis has been reported in 15% of patients treated with 3-week regimen of paclitaxel with cisplatin [1], and similar incidence of severe gastrointestinal mucositis has been reported for paclitaxel/carboplatin combination [7,20]. Serious gastrointestinal mucositis is less frequent in patients treated with weekly regimens [21]. However, some gastrointestinal symptoms are observed in most patients treated with paclitaxel/platinum combination [1,22], and manifestations of gastrointestinal mucositis have a major impact on the quality of life. In the present study, we have observed a transient increase in lactulose/monosaccharide (mannitol or xylose) ratios that returned later to pre-therapeutic levels. Similarly, sucrose/monosaccharide ratios increased during the first cycle indicating gastroduodenal mucositis. While all patients had assessment of gastroduodenal and intestinal permeability during the first chemotherapy cycle, the number of subsequent measurements was different because of the differences in the duration of therapy in individual patients. Because the numbers of measurements in each patient were different, an additional analysis was performed of the means of sequential measurements as described by Matthews et al. [14]. Parameters of intestinal permeability assessed at the end of the first or second cycle as well as the means of sequential measurement showed similar results.

Lactulose absorption, lactulose/mannitol, sucrose/mannitol and lactulose/xylose ratios during the first cycle of chemotherapy were markedly increased in patients who experienced toxicity, similarly to previous reports with other regimens [18,19,23]. Moreover, patients who experienced grade 3 or 4 toxicity during the first 6 weeks of treatment had significantly higher baseline lactulose/mannitol ratio, and serious toxicity was four times more common in patients with baseline lactulose/mannitol ratio 0.070 or above. Because of limited number of patients, no distinction was made with respect of the nature of the toxic events, and patients with both gastrointestinal and hematological toxicity were included in the analysis. Although an association between intestinal permeability and hematological toxicity could at first glance seem surprising, similar observations indicating that the dysfunction of small bowel mucosa could predispose a subject also for hematological toxicity have been reported previously [24]. An association between gastrointestinal mucositis and increased disaccharide/monosaccharide ratio has been so far reported in few studies [18,23,25], but the present study may be the first report indicating that increased lactulose/mannitol ratio could be a predictor of toxicity. However, the potential use of intestinal permeability measurements in the prediction of the toxicity of chemotherapy should be assessed in a larger prospective study.

The term intestinal permeability reflects the barrier function of bowel mucosa in separating the internal milieu from the outside environment both in an immunologic and a metabolic sense [26]. The disorders of this barrier function may be defined by altered permeability to different substances. ^51^Cr EDTA, polyvinyl pyrrolidone, or tobramycin have been used to study intestinal permeability in early studies [8], but recent studies have used differential urinary excretion of a disaccharide (lactulose or cellobiose) and a monosaccharide (mannitol or rhamnose) for the assessment of intestinal permeability. On the other hand, increased absorption of sucrose indicates damage to gastroduodenal mucosa. Physiologically, sucrose is digested by sucrase in the small intestine, and increased absorption of sucrose indicates increased permeability of gastric and duodenal mucosa [10]. There is only limited information on sucrose absorption in cancer patients. Fazeny-Dörner et al. [19] did not, in contrast to the results of the present study, observe any changes in sarcoma patients treated with the combination of ifosfamide, doxorubicin and dacarbazine. Thus, the present study may represent the first demonstration of increased gastroduodenal permeability in cancer patients treated with cytotoxic drugs.

## Conclusion

A transient increase in lactulose/monosaccharide and sucrose/monosaccharide ratios was observed in ovarian and breast cancer patients treated with paclitaxel and platinum. Increased lactulose absorption, lactulose/mannitol, sucrose/mannitol and lactulose/xylose ratios were evident in patients with grade 3 or 4 toxicity. Increased baseline lactulose/mannitol predicted serious toxicity.

## Competing interests

The author(s) declare that they have no competing interests.

## Authors' contributions

BM conceived the study, enrolled the patients, performed the statistical analysis and drafted the manuscript; RH helped with the design of the study and performed the measurements of lactulose, mannitol and xylose as well as participated in the data analysis and preparation of the manuscript; ED participated in the design of the study and patient enrolment, collected and analysed the data and helped with the preparation of the manuscript; JD participated in the enrolment of the patients and sample collection, design of the study, evaluation of clinical data and preparation of the manuscript; HK participated in data collection and analyses and revised the manuscript; and AT performed the measurements of lactulose, mannitol and xylose, and participated in the data analysis and preparation of the manuscript.

## Pre-publication history

The pre-publication history for this paper can be accessed here:


